# Consequences of Traumatic Brain Injury for Human Vergence Dynamics

**DOI:** 10.3389/fneur.2014.00282

**Published:** 2015-02-03

**Authors:** Christopher W. Tyler, Lora T. Likova, Kristyo N. Mineff, Anas M. Elsaid, Spero C. Nicholas

**Affiliations:** ^1^Smith-Kettlewell Eye Research Institute, San Francisco, CA, USA; ^2^Division of Optometry, School of Health Sciences, City University, London, UK

**Keywords:** oculomotor dynamics, vergence, binocular eye movements, convergence, divergence, traumatic brain injury

## Abstract

**Purpose:** Traumatic brain injury involving loss of consciousness has focal effects in the human brainstem, suggesting that it may have particular consequences for eye movement control. This hypothesis was investigated by measurements of vergence eye movement parameters.

**Methods:** Disparity vergence eye movements were measured for a population of 123 normally sighted individuals, 26 of whom had suffered diffuse traumatic brain injury (dTBI) in the past, while the remainder served as controls. Vergence tracking responses were measured to sinusoidal disparity modulation of a random-dot field. Disparity vergence step responses were characterized in terms of their dynamic parameters separately for the convergence and divergence directions.

**Results:** The control group showed notable differences between convergence and divergence dynamics. The dTBI group showed significantly abnormal vergence behavior on many of the dynamic parameters.

**Conclusion:** The results support the hypothesis that occult injury to the oculomotor control system is a common residual outcome of dTBI.

## Introduction

The coordination of the horizontal movements of the two eyes requires effective management of the action of the four horizontal rectus muscles (two per eye, the lateral and medial recti in each eye). It would seem that the most efficient approach to oculomotor control would be to provide independent control of the two eyes, such that each would receive the appropriate cortical signal to acquire the target as rapidly as possible wherever it might be in the visual field of that eye. Symmetric (disjunctive) eye movements between targets at different distances (known as *vergence* eye movements), however, are typically about a factor of five slower than parallel (conjunctive) eye movements at different locations at the same distance (known as *saccades*). If there was independent eye movement control of the two eyes, vergence and saccadic movements should be equally rapid, since there is no obvious evolutionary value in downregulating the speed of fixation at different distances if the capability had been available. The inference from this differential behavior is that vergences and saccades must be considered strong evidence that they are controlled by separate neurophysiological mechanisms [see Ref. (1), for review]. Here, we focus on the analysis of the normal dynamics of the weaker system, vergence, and its susceptibility to disruption by diffuse traumatic brain injury (dTBI).

### Types of vergence dynamics

In terms of the dynamic parameter of peak velocity, it is well established that the vergence system is constrained by a “main sequence” of peak velocity vs. amplitude that is functionally similar to that for saccades (2–4). The summary data of the last of these studies show a roughly linear increase in vergence velocity with amplitude up to about 2°, with a progressive saturation of the velocity function for larger amplitudes. For reference, the slope of the approximately linear portion of the main sequence for human vergence is about 4 (°/s)/° (4), compared with about 80 (°/s)/° for saccades (5) in the low-amplitude range, both declining somewhat at higher amplitudes.

Our own studies show a wide variety of vergence behaviors, even for symmetrical vergence to a large-field disparity target (6). Some subjects show patterns where both convergence and divergence match the behavior described above, while others show a range of idiosyncrasies, such as markedly slow divergence responses or slow convergence responses only, implying that the two vergence directions have separate control mechanisms, while others show slow responses in both vergence directions. The faster time courses usually had time-symmetric velocity waveforms, while the slow response waveforms were usually time-asymmetric.

### Forms of traumatic brain injury

Traumatic brain injury (TBI) is a term generally applied to cases of non-penetrating trauma to the head that results in damage to the brain. As such, it presents a diagnostic and treatment challenge, since the damage is internal to the closed head and cannot be directly assessed. Development and validation of accurate markers for the underlying pathology in TBI, and effective new approaches to treatment, are problems of high-health relevance for the large population of tens of millions of TBI sufferers. The average lifetime prevalence of disabling TBI is ~50 million, based on the current criteria used to diagnose TBI (7), which include duration of loss-of-consciousness (dLOC), duration of post-traumatic amnesia (dPTA), and patient interactions codified by the Glasgow Coma Scale (GCS).

Traumatic brain injury may be classified into focal and diffuse forms, depending on the presence or absence of an identifiable focus of damage in the brain. In the diffuse form (dTBI), damage to the neural tissue is difficult to detect even by current clinical brain-imaging protocols [e.g., Ref. (8)], although persistent symptoms may markedly affect the patients’ quality of life; even severe levels of dTBI are not reliably associated with brain-imaging signs in individual cases.

(In relation to terminology, we will use the term dTBI for the form of damage without obvious focal contusions, even though there is accumulating evidence (reviewed below) that the diffuse effects tend to be concentrated in the core brain structures. Thus, dTBI corresponds roughly to the category of mild-to-moderate (or non-penetrating) TBI, diagnosed as levels 9–15 on the GCS. However, dTBI is intended to include the additional criterion of lack of focal contusions on an MRI scan.)

### Damage to core brain structures in dTBI

Remarkably, what is generally considered to be diffuse brain trauma does, in fact, have a focal effect centered on the core brain structures, such as the basal ganglia and brainstem. Brain impacts entailing loss of consciousness are generally considered to cause diffuse axonal injury through the brain. Thus, the idea that the brainstem would be the focus of long-term damage, with specific reference of the oculomotor pathways in the upper brainstem, has neither been widely expressed nor used in diagnosis/treatment of dTBI deficits. Note, in particular, that the GCS assesses only eye opening and closing responses, and does not include any eye movement indices.

Significantly, in this context, recent studies have discovered that a high proportion of patients diagnosed with dTBI exhibit binocular vision dysfunctions, particularly deficiencies in the binocular coordination of eye movements (9–12). Up to 80% of presumed dTBI patients in these studies received a diagnosis of one or more forms of binocular dysfunction, including convergence/divergence, accommodative, and pursuit/saccade insufficiencies. Such losses of binocular coordination may result in deficits of oculomotor control and/or double vision, which have pronounced impact on the quality of life in tasks involving occupational and recreational reading, driving, estimating distance to targets in depth, tracking moving vehicles, media viewing, sports activities, etc.

**Figure 1:**
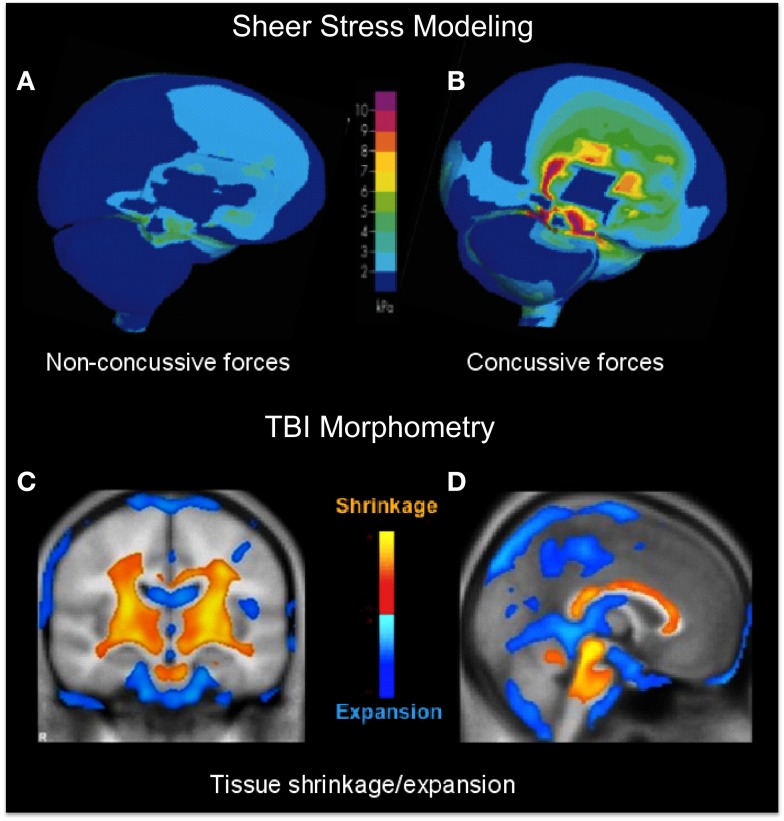
**(A,B)** Computer modeling of sheer stress forces within the brain following a strong blow to the head. The level and location predicted sheer stresses differentiate concussive **(A)** from non-concussive **(B)** injuries (13). **(C,D)** Core brain regions from a morphometric study of the regions of significant volume reduction (yellow) and increase (blue) in severe TBI patients between 8 weeks and 12 months post-injury, as compared to controls (14). Coronal **(C)** and sagittal sections **(D)** through the brain showing severe tissue shrinkage (orange coloration) in the core brain regions encompassing the basal ganglia and brainstem, respectively.

A primary indication that diffuse impacts should have a focal effect in the core brain structures comes from a study of helmet-to-helmet American football impacts by Viano et al. (13), in which the main forms of impact that produced concussion in such collisions (i.e., those meeting the definition of dTBI) were found to be oblique impacts that caused rotational acceleration, generating focal shear stresses at core brain sites localized to the corpus callosum, basal ganglia, and midbrain (Figure 1B), whereas the effects of equivalent impacts without a rotational component were far less severe (Figure 1A). In fact, it may be shown that the locus of maximal shear stress (orange coloration) matches the loci of atrophy following severe TBI (orange in Figures 1C,D) in an unrelated morphometric study (14). Significantly, although the outer cortical regions show little overall effect, the dTBI damage was focused on core brain structures including the basal ganglia (Figure 1D), and the corpus callosum and both mesencephalic and pontine levels of the brainstem (Figure 1D). Such results indicate that the “diffuse” concept of dTBI actually obscures a pronounced focus in critical control regions of the basal ganglia and midbrain, including the principal oculomotor control regions.

### dTBI and binocular eye movements

The innovative approach taken here is to consider this system as a whole, as an interconnected network of basal ganglia structures controlling all aspects of the dynamics of attentional interactions with the environment through movements of the eyes. In this sense, the approach instantiates the concept that various movements of the eyes, including the pupil and the lens accommodation, are a window into the functional status of the respective components of this complex of oculomotor control pathways. The present study focuses on the binocular aspect of oculomotor control, and specifically the control of symmetrical vergence in the median plane, which excludes any saccadic involvement.

## Materials and Methods

### Recruitment

This study involved a recruited base of 123 participants (57% female) from a non-academic population via a social media website for the normative study of oculomotor dynamics, passing the exclusion criterion of having no clinical history of brain or ocular abnormalities, including any form of strabismus or TBI events defined as involving head trauma, resulting in a loss of consciousness for a period of 5 min or more, or loss of memory of the traumatic event *per se*. All recruitment and experimental procedures in this study adhered to the Declaration of Helsinki. The study was approved by the Institutional Review Board of the Smith-Kettlewell Eye Research Institute and informed consent was obtained from all participants, none of whom withdrew from the study.

The participants were included in the analysis if they met the criteria of letter acuity of 20/40 or better in both eyes (Bailey–Lovie chart, mean LE denominator: 22 ± 5, mean RE denominator: 23 ± 6), of no visible ocular abnormalities, and of passing a random-dot stereopsis test at a disparity of 4 arcmin, consisting of reporting the quadrant and depth sign of a stereoscopically defined square of 10° on a side with a disparity of 4 arcmin. The individuals were assigned to the control group if they reported no past history of dTBI events (97 individuals with ages ranging from 19 to 62; mean age: 33.3 ± 13.3). They were assigned to the dTBI group (26 individuals with ages ranging from 21 to 64; mean age: 35.4 ± 13.8) if they reported a positive past history of one or more dTBI events characterized at levels 13–15 on the extended Glasgow Coma Scale [GCS-E; (15)] following the trauma. The participant characteristics are provided in Table 1, where the status for memory deficit on object naming, cognitive status on the clock test, and photophobia by self-report are quantified as 0 for normal, 1 for mild, and 2 for moderate. (The GCS-E categorization is not provided since evaluations at the time of the dTBI event were not available at the time of testing, and all were at level 15 when tested.) The time since the dTBI occurrence ranged from 2 months to 40 years, with a geometric mean of 4.25 years. Seventeen of the 26 reported either headaches or irritability as a result of the dTBI event. All elements in Table 1 refer to the most recent concussion, except in the column for the number of concussions previous to that one (“Previous concussions”).

**Table 1:** **dTBI participant characteristics**.

Gender	Age	Years since concussion	Concussion duration	Previous concussions	LE acuity	RE acuity	Stereo test (arcmin)	Memory deficit	Photophobia	Cognitive status	Symptoms
F	22	7	N/A	0	20/20	20/20	2	0	2	1	Headache
M	22	1	N/A	0	20/20	20/20	2	2	2	0	Dazed
F	21	1	N/A	0	20/25	20/25	2	0	2	1	Irritability
F	27	7	3 h	0	20/40	20/40	2	2	2	1	Headache
M	37	11	6 min	2	20/20	20/20	2	2	2	1	Irritability
M	38	21	N/A	0	20/16	20/16	2	0	0	1	Headache
M	25	1	N/A	1	20/20	20/20	2	2	2	1	Dazed
M	42	2	N/A	0	20/20	20/20	4	2	2	1	Headache
M	28	2	N/A	0	20/25	20/25	2	2	2	1	Headache
M	43	20	N/A	0	20/20	20/20	2	0	2	1	Dazed
M	27	10	N/A	0	20/20	20/20	2	0	0	1	Headache
M	59	2	N/A	0	20/50	20/32	2	2	2	1	
F	75	0.7	N/A	0	20/25	20/25	4	2	0	1	Balance
F	50	26	N/A	0	20/20	20/20	4	2	0	0	
F	40	1	N/A	0	20/16	20/16	2	0	2	0	Headache
M	30	8	N/A	0	20/20	20/20	2	2	2	1	Balance
M	53	35	40 h	0	20/20	20/25	2	2	2	1	Irritability
F	42	0.6	4 min	0	20/16	20/20	2	2	0	1	Irritability
F	40	0.2	3 min	0	20/20	20/25	2	2	0	1	Irritability
F	44	10	1 min	0	20/20	20/20	2	2	2	1	Headache
M	41	15	5 min	0	20/16	20/16	2	0	0	1	Headache
M	54	11	N/A	1	20/32	20/16	4	0	0	1	
M	42	1	6 min	1	20/32	20/20	2	1	0	0	Irritability
M	64	40	N/A	0	20/16	20/16	2	0	0	0	
F	52	45	20 min	1	20/20	20/16	2	2	0	0	Headache
M	32	3	2 min	2	20/20	20/125	2	2	2	1	Irritability

### Stimulus

The disparity stimulus consisted of a polarizing 3D LG monitor (LG Corporation, Seoul, South Korea), which provides chirally-distinct circularly polarized output to the two eyes when viewed with appropriate polarizing filters in front of the two eyes. The stimulus was a 20° × 40° black and white random-dot array with a central 1° cross-hair monocular fixation target. The motion of the fields could be the same or opposite for the two eyes (to provide lateral or disparity motion, respectively).

### Oculomotor procedures

**Figure 2:**
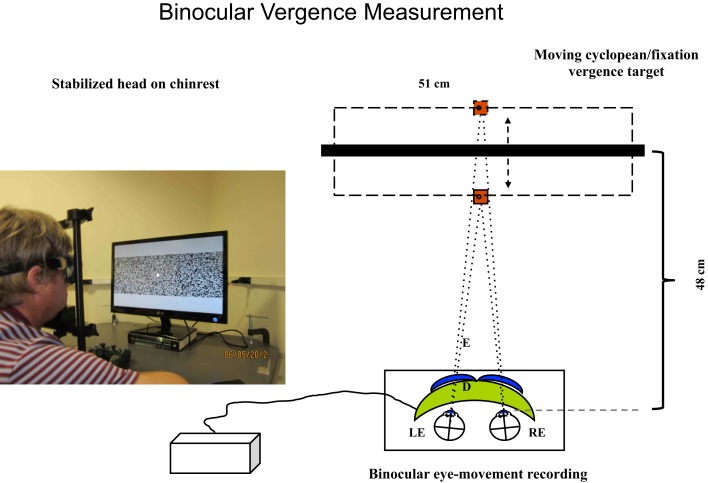
**Set-up for vergence tracking of a 40° random-dot field with the Visagraph binocular tracking system at a 48 cm viewing distance**. The thick black bar represents the light-polarizing monitor shown in the photograph, which provides oblique crossed polarized output from alternate lines of the display. The blue inserts in front of the Visagraph goggles (green) represent crossed polarizing filters. The line alternation was not visible to the participants at the 48 cm viewing distance.

Binocular eye movements were recorded with the Visagraph III (Compevo AB, Stockholm, Sweden) binocular infrared differential limbal eye tracker (Figure 2), with a sampling rate of 60 Hz and a typical noise level of 2 arcmin SD in each eye for live human recordings (as assessed from the variability during fixation periods in the most stable participants). This assessment provides a net vergence noise level of ~3 arcmin after the 4-point elliptical (third-order) smoothing applied to the eye movement traces.

### Horizontal position calibration series

To calibrate the linearity of the recorded position function, the 1° cross-hair monocular fixation target (without the random-dot field) underwent two randomized sets of horizontal position shifts over the range from −16° to 16° for each eye, with button presses indicating when fixation was accurate at each position. The full set of points was fitted with a third-order polynomial to provide a linear calibration of the horizontal position separately for each eye.

### Horizontal conjunctive tracking

Horizontal conjunctive tracking eye movements were recorded while the 40° binocular random-dot field, including the fixation target, underwent a continuous sinusoidal change of horizontal position of ±2° around the central fixation position at 0.25 Hz. This task was a designed as a control condition for the vergence tracking task (next section), using the identical stimulus but with the two fields oscillating in phase for the conjunctive tracking and in counterphase for the vergence tracking. Thus, the monocular stimuli are identical for both tasks, the only difference being the interocular phase of the sinusoidal movements.

### Horizontal disparity vergence tracking

Horizontal vergence eye movements were recorded while the 40° random-dot field, including the fixation target, underwent a continuous sinusoidal change of horizontal disparity from 8° to 12° of absolute disparity at 0.25 Hz, which is comfortably within the vergence range for normal subjects.

### Vergence tracking time-series analysis

The oculomotor position waveform for the 60 s tracking period (15 cycles) was subjected to Fourier analysis for each cycle to determine the amplitude and phase of the component at the stimulus frequency, the proportion of overall energy at the stimulus frequency (which would be 100% for perfect tracking behavior and 0.4%, or 1/240, for a pure white noise response at the 60 Hz sampling frequency). The amplitude and phase calculations were performed only on cycles in which >50% of the energy was at the stimulus frequency, to ensure that the assessment was applied when the participant was effectively engaged in tracking behavior. This analysis was performed with a sliding window of one cycle following each sample point. The fraction of cycles passing the 50% criterion was also tabulated.

### Horizontal disparity vergence steps

Binocular eye movements were recorded while the 20° × 40° noise field including the 1° fixation target that underwent 2° horizontal square-wave disparity changes every 2–3 s, with random jitter over 1 s from a uniform distribution to avoid predictability of the onset time. The minimum interval of 2 s allowed comfortable completion of 24 repeated normal vergence movements within a 60 s sequence.

### Disparity step time-series analysis

**Figure 3:**
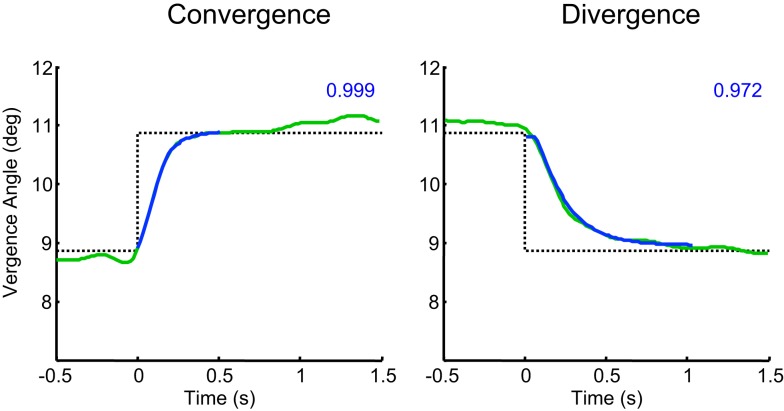
**Illustration of the fit of Eq. 1 for vergence dynamics to the fast convergence and slower divergence waveforms for one control participant**. Dotted lines: target position over time. Green curves: averaged vergence waveform specified as the difference between the left- and right- eye position waveforms. Blue curves: fitted vergence step waveform from Eq. 1, together with the R^2^ goodness of fit shown as numbers at upper right.

The vergence (left-eye minus right eye) signal waveforms were extracted from a period around the times of the instantaneous transitions of the stimulus in a window from 1 s prior to the transition to 2.5 s after the transition (see Figure 3). The sets of converging eye movements were analyzed separately from diverging eye movements. Each event response was re-zeroed by removing the mean value over the 100 ms preceding the transition. To exclude outliers, non-representative individual responses were excluded from the analysis by iteratively removing responses whose mean squared error over time was beyond 2 SD of the mean error across responses. (In no case were more than 2 of the 12 responses excluded under this procedure.)

To quantify the vergence dynamics, the average convergence and divergence waveforms were fitted with a canonical time waveform *v*(*t*) consisting of a two-component cosine + gamma function fit:

(1) v(t)=G(t)+γ(t)

where

$$\begin{array}{rcll} G\left(t\right) & = & \left\{\begin{array}{cc} 0 & ,t<\delta_{1}\\ \frac{A_{1}}{w}\left[t-\delta_{1}-\frac{w}{2\pi }\sin\left(\frac{2\pi }{w}\left(t-\delta_{1}\right)\right)\right] & ,\delta_{1}\leq t\leq \delta_{1}+w\\ A_{1} & ,t>\delta_{1}+w \end{array}\right. & \\ \gamma \left(t\right) & = & \left\{\begin{array}{cc} 0 & ,t<\delta_{2}\\ A_{2}\int_{\delta_{2}}^{t}\frac{1}{\beta^{\alpha }\Gamma \left(\alpha \right)}{\left(\text{T}-\delta_{2}\right)}^{\alpha -1}\;\exp\left(-\frac{\text{T}-\delta_{2}}{\beta }\right)d\text{T} & ,t\geq \delta_{2} \end{array}\right. & \end{array}$$

where *t*, T are time variables, *A*_1_, *A*_2_ are scaling factors, δ_1_, δ_2_ are delays, *w* is the period of the cosine, β is the exponential time constant, and α is the order of the gamma function.

The fit of this equation, as characterized for one example in Figure 3, allowed the quantification of five parameters of the vergence dynamics, separately for the convergence and divergence directions: onset latency, duration, amplitude, peak velocity, and temporal asymmetry. Duration was defined as the time between the 5 and 95% points of *v*(*t*). Peak velocity was defined for the peak of its derivative. Temporal asymmetry is defined in the following section.

### Temporal asymmetry

Temporal asymmetry of the velocity trace was defined by computing the ratio of the post-peak area minus the pre-peak area to the total area of the vergence interval defined from the velocity trace. In principle, this temporal asymmetry index has a value of 0 for a time-symmetric waveform and a value of 1 for a pure exponential waveform. In practice, the smoothing applied to the waveform reduces the maximum value for the pure exponential response after the filtering of the waveforms, so we defined a normalized temporal asymmetry index (γ) as the ratio of the empirical temporal asymmetry index to the theoretical temporal asymmetry index for a filtered exponential decay. (Note that a waveform with an asymmetry sharper than the exponential form could have γ > 1.0).

### Statistical analysis

The statistical analyses were performed by *t*-tests. Unless otherwise noted, significant results are reported at level of *p* < 0.01.

## Results

### Vergence tracking deficits

**Figure 4:**
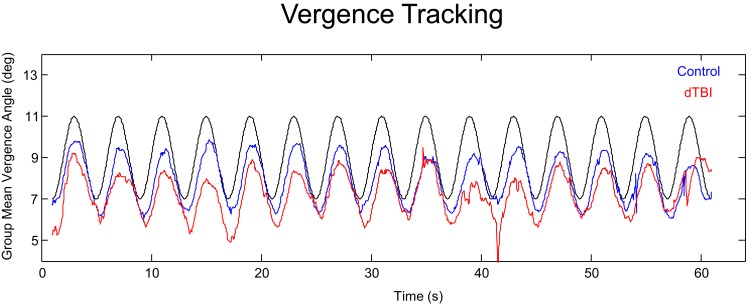
**Average vergence (disjunctive) tracking waveform for the control (blue) and dTBI (red) populations to the target disparity (black curve)**.

An overview of the vergence tracking behavior is provided by averaging the sinusoidal tracking performance separately for the control and dTBI groups in relation to the target disparity variation (black curve in Figure 4). The average waveform for the control group (blue curve in Figure 4) shows (a) that the amplitude of vergence tracking was reduced relative to the target disparities and (b) that there was a net vergence error to diverge accurately to the far target disparity (8°) but not to converge fully to the near target disparity (12°). The dTBI population (red curve in Figure 4) shows an amplified version of the same tendencies. In fact, the mean vergence angle is reduced close to 0, although the tracking amplitude was similar to that for the control group. The results of this and the following analyses are tabulated in Table 2. Comparisons significant at *p* < 0.01 are highlighted in yellow.

**Table 2:** **Sinusoidal tracking parameter values and statistical analysis**.

	Mean target vergence (deg)	Mean vergence (deg)	Mean vergence error (deg)	Target vergence amplitude (deg)	Mean vergence amplitude (deg)	Normalized vergence amplitude (deg)	Mean phase lag (ms)	Proportion of good cycles (%)
Controls	9	8.02	−0.98	4	3.34	0.84	222	33
SEM			0.12			0.019	17	5
dTBI	9	8.73	−0.27	4	3.46	0.87	257	20
SEM			0.24			0.022	197	8
*P*			< 0.01			NS	NS	NS
Specificity (%)		91			91		92	93
Predicted		9			9		8	7
Specificity (%)								
Sensitivity (%)		17			26		9	26
F(SEM)			2.83			1.26	11.6	
*p*			<0.05			NS	< 0.01	

**Figure 5:**
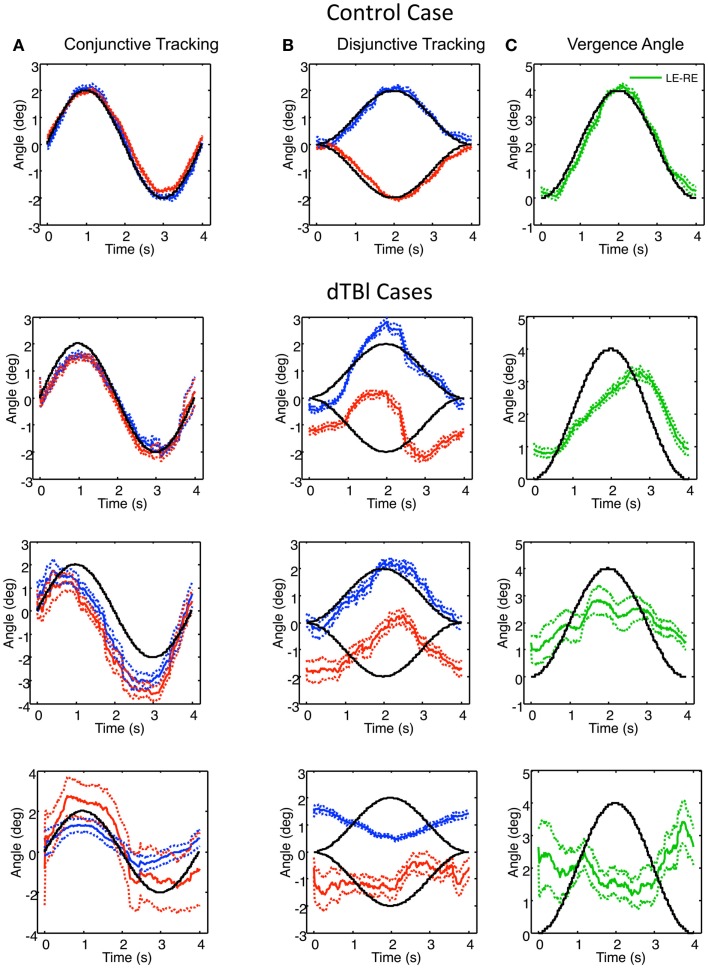
**Example of control (top) and three dTBI cases of sinusoidal tracking performance**. **(A)** shows the average left (blue) and right (red) performance in tracking a 4° amplitude sinusoidal target movement over 12 cycles (black curve). **(B)** uses the same conventions for tracking opposite direction 2° amplitude sinusoidal target movements in the two eyes. **(C)** shows the difference function (green curve coding the vergence tracking performance). Dotted lines show ±1 SEM around the mean over 12 cycles. Note the highly irregular and diverse tracking behavior in the dTBI cases.

Examples of individual eye movement analysis for the sinusoidal tracking paradigm are shown in Figure 5, for a control individual (top row) and for three dTBI sufferers (lower rows). Figure 5A shows the average cycle analysis of the horizontal sinusoidal position signal for *conjunctive tracking* by the two eyes. Figures 5B,C show the horizontal vergence tracking by the two eyes of the random-element plane of Figure 2 moving sinusoidally in counterphase to generate a sinusoidal depth motion. Figure 5B shows the individual *disjunctive tracking* movements of the two eyes and Figure 5C shows the net *vergence tracking* signal formed from the difference between the two eye positions in Figure 5B. In each case, the target movements to be tracked are shown as the black traces.

For the control case (upper row, Figure 5), the two eyes’ *conjunctive tracking* movements match the movement of the target position in Figure 5A well and in excellent synchrony with each other. In Figure 5B, the *disjunctive tracking* movements also show an excellent match to the disparity amplitude and waveform to the stimulus movement in each eye, providing an excellent match to the *vergence tracking* signal in Figure 5C except for a small phase lag of about 0.1 s.

In the uppermost dTBI case in Figure 5, the horizontal *conjunctive tracking* is similar to the control, while the *disjunctive tracking* is a consistent mixture of smooth tracking and saccades that deviates radically from the target trace. The disjunctive tracking is actually dominated by a conjunctive similarity between the two eyes’ trajectories, though with a minor difference between them. (Note the small error ranges on either side of the mean traces, indicating how stable this jerky behavior is across repeats, however.) The difference function reveals that the *vergence tracking* is adhering relatively closely to the target disparity, though significantly delayed.

The center dTBI case in Figure 5 shows somewhat similar characteristics, although the *conjunctive tracking* is substantially advanced in phase relative to the target. The *disjunctive tracking* of the two eyes are again largely conjunctive in nature, with any difference barely noticeable. Yet, the *vergence angle* (interocular difference) function reveals that there is a reportable vergence tracking of about half the target amplitude, though with much larger error than in the control case.

In the third dTBI case in Figure 5, even the *conjunctive tracking* is heavily disrupted, with jerky behavior, large differences between the eyes and wide error ranges, indicating inconsistent behavior from trial to trial. The right-eye variability is again large, although the left-eye shows a consistent tendency of anti-tracking, as though it is driven by the right-eye signal. The *disjunctive tracking* behavior shows inversion of the left-eye response, though with reduced amplitude, and a much noisier response for the right eye. These combine to produce a *vergence tracking* response with little adherence to the target disparity, and may be regarded as essentially noise, or perhaps even inverted on the basis of the inverted left-eye response.

To quantify these results, the average response to the sinusoidal disparity stimulus may be encoded by the following parameters: amplitude and phase delay in terms of absolute response lag, together with their SEM over cycles, and a parameter for the goodness of fit to the sinusoidal waveform. In order to avoid artifacts due to blinks and inattention, the analysis was based only on cycles in which the goodness of fit to the sinusoidal waveform was better than a correlation value of 0.9 (based on a 1-cycle moving average).

**Figure 6:**
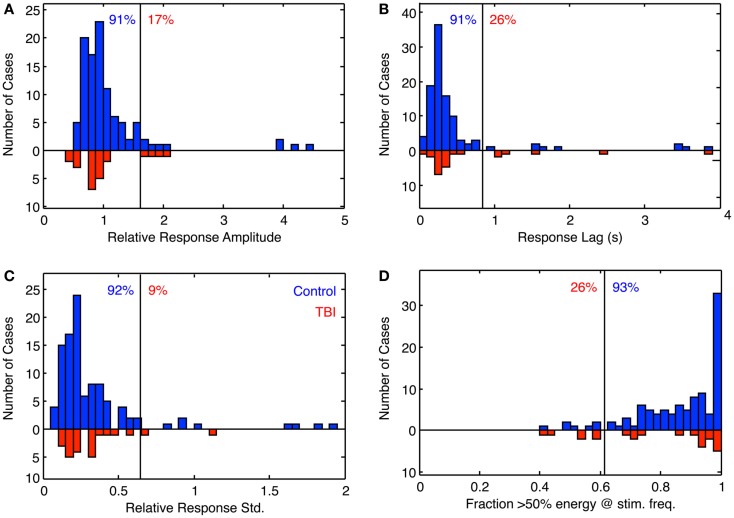
**Histogram of the fitting parameters for sinusoidal fit to sinusoidal vergence tracking**. Blue bars: controls. Red bars: dTBI individuals (on inverted frequency axis). **(A)** Relative response amplitude (2° vergence demand). **(B)** Response lag(s). **(C)** SD relative to response amplitude over 15-cycle period (based on 1-cycle moving average). **(D)** Fraction of cycles with more that 50% of the response energy at the stimulus frequency (based on 1-cycle moving average). Vertical line: criterion line at first bin beyond 90% of distribution. Blue number: percent of control distribution below criterion line. Red number: percent of dTBI distribution above criterion line.

The full results for these four parameters are shown in Figure 6 for the population of 97 control (blue bars) and 26 dTBI individuals (inverted red bars). The results are surprising in many respects. The tracking amplitude (Figure 6A) peaks close to a gain of 1, but it has a long tail to the high side implying that a small proportion of control individuals track (sinusoidally) at substantially larger amplitude than the disparity demand. Since by visual inspection this and the other histograms in Figure 6 are substantially skewed, they were fitted with a two-component model combining a Gaussian distribution with a the gamma distribution function (*x^(k^*^−^*^1)^* × *e*^−^*^x/σ^*), where *k* and *σ* are constants, which has a long tail for small *k*.

The fit to the phase distribution (Figure 6C) is a special case, since it is not limited to a >0 phase, and since it is a circular variable that should be fitted to the Rice distribution if the noise was inherently Gaussian. However, since it has a small SD relative to the full phase cycle, it has been fitted with the gamma distribution with phase lag as a free parameter ((*x* − *x_o_*)^(^*^k−^*^1)^ ×* e−*^(^*^x−xo^*^)/^*σ*), where *x* is phase lag, to avoid biasing the peak of the distribution while allowing for an asymmetry of the distribution.

The goodness of fit distributions (Figure 6D) show that all individuals had good-quality sinusoidal tracking waveforms (correlations > 0.9) for at least 40% of the 1-min tracking period, so the other indices had a firm foundation in this respect.

The fit for the amplitude distribution gives a peak-to-peak value of 3.34° ± 0.076°, implying that the typical behavior is to track at a significantly lower disparity amplitude than the stimulus demand (which is normalized to 1). However, 17% of the population show amplitudes >1.5 (3°), forming the long tail to the right of the main peak, and this result cannot be attributed to simply noisy tracking behavior because the amplitude analysis is based only on good-quality tracking cycles.

The peak phase lag was 222 ± 17 ms, implying that the normal behavior is to track the target with a neural processing delay typical for unpredictable saccades. Here, the surprise is that virtually all cases are showing a phase lag despite the fact that the stimulus disparity was entirely predictable after the first cycle. The asymmetry in the fitted gamma function implies that a small proportion of the control group have atypically long tracking lags (see Discussion). The relative SD (coefficient of variation) was 0.16, implying a good consistency of the vergence tracking behavior over cycles, and about 1/3 of the population achieve a tracking consistency of >98%.

The same set of analysis was performed for the dTBI group (red bars, inverted ordinate in Figure 6). The mean amplitude was 3.080° ± 0.096°, implying that the amplitudes are significantly lower than in the control group. Similarly, the mean response lag was 257 ± 19 ms, which is significantly slower than the controls. A variability analysis shows that the phase lags are also significantly more variable for the dTBI group than the control group (see Table 2).

The data can be also used for a sensitivity/specificity analysis, shown as the proportions at the top of each graph. The specificity was picked as the first bin division in the histogram above the 90% level (91, 91, 92, and 93%, respectively, for the four graphs in Figure 6). These criteria predict that, if the dTBI values were drawn from the same population of oculomotor performance characteristics as the control, they should show the complement of these values as the proportion of cases falling above the criterion level, namely 9, 9, 8, and 7%, respectively (see Table 2). In fact, the values for this proportion of dTBI cases falling above the respective criterion levels are 17, 26, 9, and 26%. Thus, the vergence tracking task is showing abnormalities in about a quarter of the dTBI cases for a criterion level that would be expected to show <10% of such cases.

### Step vergence deficits

**Figure 7:**
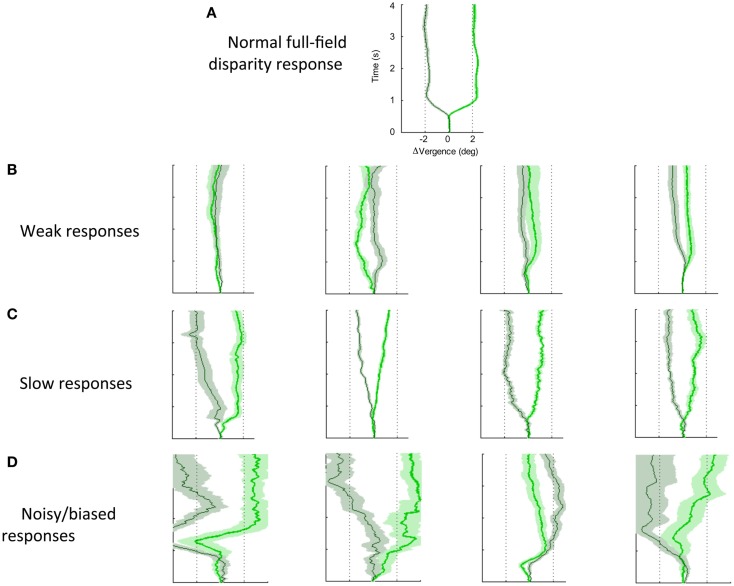
**Effects of dTBI on vergence dynamics**. **(A)** Typical example of normal convergence (light green line) and divergence (dark green line) responses averaged over 12 repeats, with time running vertically and disparity on the horizontal axis. Color band around each line indicates SE of the average functions. **(B)** Four examples of weak vergence responses in dTBI, with amplitudes <25%. **(C)** Four examples of slow dTBI vergence responses, reaching approximately full amplitude after 1–2 s. **(D)** Four examples of noisy and biased dTBI vergence responses.

Figure 7 shows a variety of the deficits in vergence dynamics from the group of 26 dTBI sufferers, relative to one example from a non-dTBI individual (Figure 7A). The majority this group showed notably abnormal vergence dynamics, which fell largely into the three forms depicted in Figure 7; Figure 7B weak, Figure 7C slow, and Figure 7D noisy responses that are biased to start in the same direction regardless of the disparity change (despite all participants exhibiting verified fine stereopsis). This analysis of the variety of human vergence responses thus contributes substantially to the understanding of the deficits in the oculomotor control mechanisms resulting from dTBI.

### Quantitative analysis of vergence dynamics

The step vergence dynamics were quantified on five parameters, separately for the 2° convergence and divergence directions: onset latency, duration, amplitude, peak velocity, and temporal asymmetry. The average values for these parameters are tabulated in Table 3. Note that, in some cases, the responses were too variable to justify such quantitative analysis; responses were excluded if the average SD over the 12 repeats exceeded 1°. The general picture from this table is that convergence and divergence parameters are largely similar, although onset latencies are significantly longer for divergence than convergence responses in both groups, and the durations are significantly longer for divergence in the control group. Comparisons between the control and DTBI groups are indicated in bold font for the means (t values) and SDs (F values).

**Table 3:** **Values and significances for Gaussian model fits**.

Parameter	Amplitude (deg)		Onset latency		Peak velocity		Duriation (s)		Asymmerty	
Vergence Dir	Conv	Div	Conv	Div	Conv	Div	Conv	Div	Conv	Div
Control mean	2.03	1.53	0.24	0.21	7.96	5.44	0.38	0.45	0.14	0.16
Control sigma	0.44	0.41	0.03	0.08	1.25	2.06	0.03	0.04	0.10	0.10
Control t(Conv-Div)		**8.50**		**3.23**		**10.73**		**13.46**		**1.50**
dTBI mean	1.60	1.97	0.20	0.30	5.77	3.02	0.38	0.41	0.09	0.23
dTBI sigma	0.27	0.31	0.02	0.04	1.25	1.02	0.09	0.04	0.09	0.18
dTBI t(Conv-Div)		**9.33**		**22.30**		**17.49**		**3.50**		**7.09**
t(Control-dTBI)	**6.28**	**5.96**	**7.06**	**8.05**	**7.91**	**8.45**	**0.39**	**4.49**	**2.43**	**1.88**
Control Var SD	0.14	0.15	0.03	0.07	0.65	0.79	0.04	0.08	0.11	0.12
dTBI Var SD	0.22	0.19	0.03	0.05	0.59	0.61	0.09	0.09	0.12	0.12
Control F(Conv-Div)		**1.18**		**5.44**		**1.47**		**3.22**		**1.11**
dTBI F(Conv-Div)		**0.74**		**2.78**		**1.07**		**1.02**		**1.05**
F(Control-dTBI)	**2.53**	**1.60**	**1.00**	**0.51**	**0.82**	**0.60**	**3.99**	**1.27**	**1.06**	**1.00**

**Figure 8:**
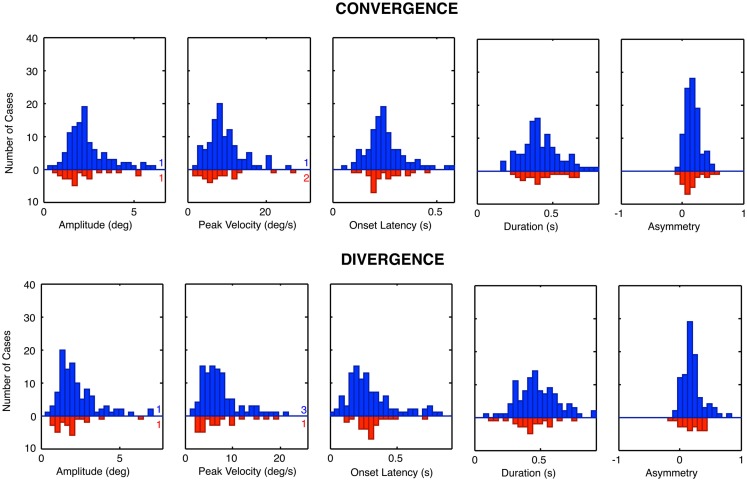
**Parameter distributions for the five parameters of vergence dynamics average values over the 12 repeats of the convergence and divergence step responses for each individual for the control group (blue bars, upward axis range) and dTBI group (red bars, downward axis range)**. Note spread of tail to the high side in many of the distributions.

The group distribution parameters for the dTBI subgroup are mostly similar to those of the controls, except for the case of the amplitude variances (which are significantly larger for the dTBI group than for the control group for both convergence and divergence responses) and the peak velocities (which are consistently lower for both convergence and divergence responses, but do not reach the criteria for significance). We therefore turn to a more detailed analysis of the parameter distributions plotted in Figure 8, in order to determine the underlying diagnostic state of affairs, which shows that the extended tails on many of the distributions are diluting the significance of the distribution means.

### Vergence distribution model fits

**Figure 9:**
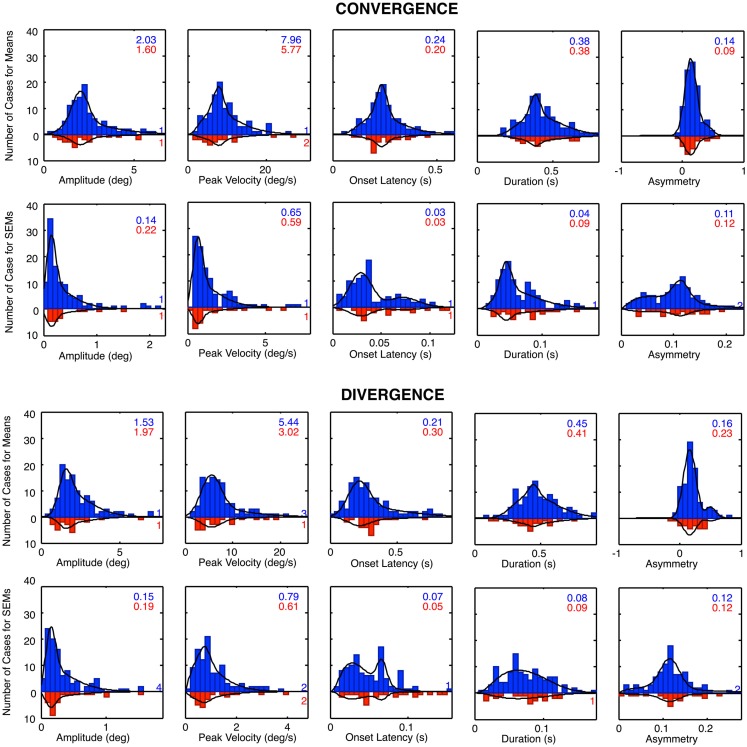
**Two-component Gaussian-gamma fits (black curves) to the distributions of the mean values and the SEM values of the convergence and divergence step responses in the control groups (blue bars) and dTBI group (red bars)**. Inset numbers specify the peak value for the Gaussian component for each group. Numbers near axis at right indicate the number of cases that exceeded the axis range for each group.

The histograms of Figure 8 reveal that many of the control vergence parameters have distributions that deviate substantially from the normal bell-curve shape (not shown), having long tails or a second peak beyond the main peak. Many of the distributions differed significantly from the Gaussian fit at *p* < 0.01, and the average chi square across the six distributions showed that they were significantly non-Gaussian at this level for both the convergence and the divergence sets. We therefore fitted the distributions with a two-component model consisting of a normal Gaussian function summed with a three-parameter gamma function that could allow for long tails and/or a second peak (Equation 1; black curves in Figure 9). Generally, the two-component model fitted the control distributions accurately with no significant deviation, at the criterion of *p* > 0.1. The only exception was the peak velocity time for the divergence responses, whose fit reached *p* = 0.03, but that did not quite pass the criterion for a significant deviation from the fit after correction for multiple tests, which was *p* < 0.01.

A second form of analysis of the vergence performance is the repeatability of the vergence dynamics over many trials. Here, again the distributions differed significantly from the Gaussian fit at *p* < 0.01 for many of the parameters, and the average chi square across the five distributions was significantly non-Gaussian for both the convergence and the divergence sets. We therefore fitted the repeatability distributions with the same two-component model, which gave satisfactory fits with non-significant deviations for about half of the convergence and divergence parameters, leaving significant deviations for the remainder. These were considered adequate characterizations of the primary features of the repeatability distributions, however, as these are not the main focus of the analysis.

For the mean distributions, ~80% of the area was accounted for on average by the predominant Gaussian component, with the remaining ~20% of the extended tail region fitted by the gamma component. Since the Gaussian is the expected distribution for data perturbed by multiple noise sources, according to the Central Limit Theorem, we take this result as evidence that the Gaussian component represents the error distribution of the normal population on each parameter and that the second component represents a subpopulation with some distinct deviation from normal functioning. One hypothesis for the source of such a deviation is the occurrence of non-reported dTBI events, which might have long-lived consequences going back as far as a birth trauma (since there is evidence for long-lasting effects of dTBI in the data for the dTBI group). This interpretation implies that the Gaussian component is the best estimate of the normal behavior for the control group, and that the Gaussian component for the dTBI group captures the predominant behavior for this subgroup (with the range of diverse forms of damage captured by the gamma component for both groups). The analysis of abnormality for the dTBI group is consequently evaluated in relation to the Gaussian component of the control group. We have therefore tabulated the mean values for the Gaussian component for the control group for comparison with the values in the dTBI group.

Table 3 reports comparison of the mean parameters of the Gaussian component of the distribution fits, both between convergence and divergence and between the control and dTBI groups. Significant values are indicated by color coding of the cell. Yellow denotes a significant difference in either direction between the convergence and divergence parameters. Blue denotes a significant degradation in performance for the dTBI group relative to controls (where an increase in variability is treated as a degradation). Pink codes for a corresponding significant improvement in performance. Note that a degradation in performance implies different effects on different indices. For the amplitude and peak velocity indices, degradation is implied by a reduction in the index value. For the onset latency, duration and temporal asymmetry (γ) indices, on the other hand, degradation is generally implied by an increase in the index value.

Table 3 incorporates several analyses. Within the control group, it provides a comparison of the mean Gaussian fits for divergence vs. convergence (yellow highlighting), showing that the divergence values were significantly degraded (i.e., lower or higher than for the controls, respectively, as specified in the previous paragraph) for the amplitude, peak velocity, and duration parameters, implying a general weakness for the divergence system in normal individuals of the order of 20%. Interestingly, however, the average value was shorter for onset latency, implying that the divergence responses were initiated more quickly than the convergence responses.

For the dTBI group, Table 3 shows that the mean values were significantly degraded (blue highlighting) relative to those of the control group for the amplitude and peak velocity parameters for convergence movements and for onset latency and peak velocity parameters for divergence movements (two-tailed *t*-tests at *p* < 0.01). Remarkably, the convergence/divergence performance for the dTBI group were significantly enhanced (pink highlighting) relative to controls for the onset latency parameter for convergence and for the amplitude and duration parameters for divergence.

This table also provides an analysis for the variability of the parameters over trials, which was significant higher for the dTBI than for the control group for the amplitude and duration parameters in the convergence (but not the divergence) direction. The lack of an effect in the divergence directions is partly due to the significant tendency for the variabilities for the control group to be higher for divergence than convergence movements (significant for the onset and duration parameters). (Note that these variabilities are analyzed on the one-tailed hypothesis that the variability is expected to be higher for the dTBI than the control group.)

In summary, the Gaussian model fits provide strong evidence for notable differences in vergence eye movement dynamics for dTBI sufferers relative to the control population, building on the basic information of significant differences between divergence and convergence movements in the control population itself. Most of the differences are in the direction of weaker responses in the dTBI population.

### Correlation analysis

As a final form of analysis, correlations were run for each of the step disparity convergence and divergence parameters of Table 3 with the two demographic parameters of age and years since concussion in the mTBI group. None of these correlations were individually significant at *p* < 0.01 (uncorrected), implying that they would be even further from significance if an appropriate correction level was employed for the 20 applications of the significance test. Thus, we can conclude that there was no significant variance introduced by the wide range of ages or time since the concussion event.

## Discussion

### Controls

The average vergence tracking waveform for the control group showed a mean vergence error of about 1° less than the target vergence angle of 9°, implying that the vergence was relaxed somewhat behind the screen (at the 48 cm viewing distance).

The control convergence/divergence comparisons in the controls are of interest in assessing the dynamics of the vergence control system in its near-field operating range (6). The present study extends the result from our previous study to a larger sample, verifying that the divergence values were significantly degraded for the amplitude, peak velocity, and duration parameters, and implying a general weakness for the divergence system in normal individuals of the order of 20%. Interestingly, however, the average divergence value was significantly shorter for onset latency, implying that the divergence responses were initiated more quickly than the convergence responses, perhaps implying a dependence on the absolute disparity of the starting position.

The long tails on many of the normal distribution functions imply that they are not well described by Gaussian distributions, which are the asymptotic form to be expected from the combination of many independent sources of noise (regardless of whether the individual sources themselves have Gaussian distributions). The long tails may thus be interpreted to imply that some proportion of the individuals categorized as normal should be suspected of exhibiting abnormal oculomotor dynamics on the respective indices. In the context of the deficits shown by the dTBI sufferers, it needs to be asked whether the clinical history could have missed some TBI incidents in these cases. Although our clinical history asked about all TBI incidents in the individuals’ past, it is unlikely that the history would unearth all TBI incidents in a person’s life, particularly those due to birth trauma or falls during infancy. The analysis thus suggests that the long tails of the “control” distribution functions represent an occult subpopulation of dTBI cases.

In the convergence/divergence comparison in the controls, the primary result is the combined weakness in the divergence response relative to the convergence responses on all parameters except temporal asymmetry. The lack of a significant difference for the temporal asymmetry parameter implies that the general weakness is not attributable to a change in the operating principle (active vs. passive) between the two directions of movement as indexed by a change in the waveform of the saccadic responses. Evidently, there is just a net tendency toward a weaker response in the divergence direction on a population basis, although the analysis of Tyler et al. (6) made clear that a good proportion of individuals had no significant difference between the two vergence directions.

### dTBI group

For the dTBI group, the mean values of most parameters were significantly weaker than those of the control group for both convergence and divergence movements. The mean vergence angle was reduced by 1.8° relative to the 9° vergence angle of the screen, implying that the dTBI group exhibiting a further degree of convergence relaxation on average, although the tracking amplitude was similar to that for the control group. This result corresponds to a quantification of the condition known as “convergence insufficiency” that is typically associated with dTBI. We emphasize that all the dTBI individuals had normal stereoscopic vision for the random-dot depth discrimination targets of our screening test, so this difference is not attributable to a sensory weakness. Remarkably, however, the convergence/divergence comparisons for the dTBI group go in the reverse direction from the controls for the amplitude and onset latency parameters, although there is no obvious reason why this should be the case. The variability of the parameters over trials was also significantly higher for the dTBI than for the control group for the amplitude and duration parameters in the convergence direction.

The sinusoidal vergence tracking data show a significant proportion of deficits in the dTBI, with up to a quarter of cases showing weakness on three of the vergence tracking parameters, where only about 7–9% should be expected from the criteria set for the control distributions. It thus should be worthwhile to include this form of tracking behavior in a test for binocular deficits in dTBI cases.

The reduced amplitudes of convergence responses in the dTBI subgroup is not surprising in light of the higher proportion of convergence insufficiency reported in clinical studies of this population (9, 10, 16, 17), although those studies focus on the extremes of the convergence range whereas for the present study the amplitude of small (2°) vergence responses were well within the functional range of normal vergence behavior. The dTBI conditions drop the peak of the amplitudes to about 80% for convergence. Remarkably, the divergence amplitude shows the opposite effect for the dTBI group, with a significant increase of 24%, implying a recovery of divergence to the typical control characteristics for convergence. However, it is noteworthy that this reversion occurs in combination with a significant further reduction in peak velocity, suggesting that it represents a slowing of the divergence system, allowing it to progress to full amplitude as a result.

One hypothesis for the effect of dTBI could be that it might tend to knock out the active drive of the vergence response in one direction and convert it to a passive response. If this were to occur, an index of its occurrence might be the switch from a time-symmetric waveform to a high-asymmetry exponential waveform corresponding to relaxation process (6). However, the proportion of asymmetric responses was high in both groups and showed no difference in the dTBI population relative to the controls, invalidating the passive-drive explanation for the exponential waveform. On the other hand, the exponential waveform is also compatible with an active feedback error-minimization process in which the vergence velocity is proportional to the target disparity error, which may account for its prevalence in the non-dTBI population.

## Conclusion

This study shows that the variety of human vergence dynamics contributes substantially to the understanding of the oculomotor control mechanisms underlying the generation of these movements, and their susceptibility to mild TBI. A large proportion of the dTBI group showed abnormal vergence behavior on one or more of the dynamic parameters. The results suggest that occult injury to the oculomotor control system is a common residual outcome of dTBI. Severe brain injury is often visible by structural brain imaging, such as X-radiography or magnetic resonance imaging (MRI), but milder effects that are invisible to these techniques may nevertheless cause substantial oculomotor disruptions. Effective treatment of these oculomotor problems will require accurate diagnosis of the source of the problem.

## Conflict of Interest Statement

The authors declare that the research was conducted in the absence of any commercial or financial relationships that could be construed as a potential conflict of interest.
